# Modeling interactions between Human Equilibrative Nucleoside Transporter-1 and other factors involved in the response to gemcitabine treatment to predict clinical outcomes in pancreatic ductal adenocarcinoma patients

**DOI:** 10.1186/s12967-014-0248-4

**Published:** 2014-09-10

**Authors:** Francesca Tavano, Andrea Fontana, Fabio Pellegrini, Francesca Paola Burbaci, Francesca Rappa, Francesco Cappello, Massimiliano Copetti, Evaristo Maiello, Lucia Lombardi, Paolo Graziano, Manlio Vinciguerra, Fabio Francesco di Mola, Pierluigi di Sebastiano, Angelo Andriulli, Valerio Pazienza

**Affiliations:** Gastroenterology Unit, I.R.C.C.S. “Casa Sollievo della Sofferenza” Hospital San Giovanni Rotondo (FG) Italy, viale dei Cappuccini n.1, San Giovanni Rotondo, FG 71013 Italy; Unit of Biostatistics I.R.C.C.S. “Casa Sollievo della Sofferenza” Hospital San Giovanni Rotondo (FG) Italy, viale dei Cappuccini n.1, San Giovanni Rotondo, FG 71013 Italy; Unit of Biostatistics, DCPE, Consorzio Mario Negri Sud, Santa Maria Imbaro, CH Italy; Euro-Mediterranean Institute of Science and Technology, Palermo, Italy; Department of Experimental Biomedicine and Clinical Neuroscience, University of Palermo, Palermo, Italy; Oncology Unit I.R.C.C.S. “Casa Sollievo della Sofferenza” Hospital San Giovanni Rotondo (FG) Italy, viale dei Cappuccini n.1, San Giovanni Rotondo, FG 71013 Italy; Pathology Unit I.R.C.C.S. “Casa Sollievo della Sofferenza” Hospital San Giovanni Rotondo (FG) Italy, viale dei Cappuccini n.1, San Giovanni Rotondo, FG 71013 Italy; University College London, Institute for Liver and Digestive Health, Division of Medicine, Royal Free Campus, London, UK; Division of Surgical Oncology “SS Annunziata” Hospital, Chieti, Italy

**Keywords:** Pancreatic ductal adenocarcinoma, hENT1, CHOP, MRP1, DCK, RECPAM

## Abstract

**Background:**

Pancreatic ductal adenocarcinoma (PDAC) is an extremely aggressive malignancy, characterized by largely unsatisfactory responses to the currently available therapeutic strategies. In this study we evaluated the expression of genes involved in gemcitabine uptake in a selected cohort of patients with PDAC, with well-defined clinical-pathological features.

**Methods:**

mRNA levels of *hENT1*, *CHOP*, *MRP1* and *DCK* were evaluated by means of qRT-PCR in matched pairs of tumor and adjacent normal tissue samples collected from PDAC patients treated with gemcitabine after surgical tumor resection. To detect possible interaction between gene expression levels and to identify subgroups of patients at different mortality/progression risk, the RECursive Partitioning and Amalgamation (RECPAM) method was used.

**Results:**

RECPAM analysis showed that *DCK* and *CHOP* were most relevant variables for the identification of patients with different mortality risk, while *hENT1* and *CHOP* were able to identify subgroups of patients with different disease progression risk. Conclusion: *hENT1*, *CHOP*, *MRP1* and *DCK* appear correlated to PDAC, and this interaction might influence disease behavior.

## Introduction

Pancreatic ductal adenocarcinoma (PDAC) is the fourth leading cause of cancer-related death [1,2] with a poor prognosis due to the early undetectable symptoms and the lack of effective treatment which are responsible for the high lethality [3,4]. There is an urgent need for additional biomarkers which can predict the PDAC onset, or for implementing new effective therapeutic strategies. Gemcitabine is considered the standard of care for the treatment of patients with locally advanced and metastatic PDAC, with either curative or palliative intent [5,6]. Gemcitabine was synthesized in early 1980s and chemically is a nucleoside analogue (similar to cytosine) which displays two fluorines on the carbon 2′, instead of the hydrogen atoms, conferring it tumor growth arrest properties.

Recently, research efforts were boosted in order to find biomarkers effective into predict the clinical benefits of gemcitabine in PDAC [7,8]. Among the factors involved in the gemcitabine response pathway, human equilibrative nucleoside transporter 1 (*hENT1*) has been reported as the main mediator of gemcitabine uptake across plasma membranes [9], and it has been associated to gemcitabine-dependent effects in several *in vitro* and *in vivo* studies [10-14]. The deoxycytidine kinase (*DCK*), represents another key enzyme involved in gemcitabine phosphorylation/activation, and it has also been implicated in gemcitabine resistance [15], as well as the multidrug resistance-associated protein 1 (*MRP1*) which is involved in chemotherapy resistance in human pancreatic cancer [16,17]. Further, CCAAT-enhancer-binding protein homologous protein (*CHOP*) represents a stress-induced transcription factor involved not only in the cell cycle and apoptosis [18], but also in the transcriptional down-regulation of *hENT1* expression [19].

Herein we analyzed potential changes in the expression levels of *hENT1*, *DCK*, *MRP1* and *CHOP*, as are the main factors involved in the gemcitabine response pathway and they are involved in gemcitabine uptake and activation, in biopsies of PDAC patients in order to find possible associations between the mutual expression levels of these genes and clinical pathological features in a selected and well characterized cohort of patients with PDAC.

## Material and methods

### Clinical samples

The training cohort included tissues specimens collected from 26 patients with pancreatic ductal adenocarcinoma (PDAC). These patients were selected among a total of 76 subjects, with final pathological diagnosis of PDAC, who underwent pancreatic resection and at our hospital IRCCS “Casa Sollievo della Sofferenza” San Giovanni Rotondo (Italy), between March 2008 and May 2014. The inclusion criteria included: availability of matched pairs of tumor and adjacent normal tissue sample (54 out of 76), adjuvant chemotherapy with gemcitabine administered after complete resection of pancreatic cancer in absence of neo-adiuvant treatments (26 out of 54), and complete follow-up data, including either clinical examination of CA 19–9 serum marker and monitor of response to treatment at regular intervals (26 out of 26). In details, all the 26 patients fulfilling these criteria, weekly received gemcitabine at a dose of 1,000 mg/m2 for 7 weeks as an induction phase. After this phase, CA 19–9 levels were elevated in 10 patients. This patients underwent to computed tomography scan that showed visceral metastases (liver, mesenteric, lung etc.), therefore were evaluated for a first line treatment. The remaining patients with normal CA 19–9 levels, entered the chemoradiotherapy phase of the treatment. In five patients, gemcitabine 400 mg/m2 weekly × 3 every 28 days for 2 cycles, and concurrent radiotherapy, for a total dose of 50.4 Gy in 28 fractions were prescribed. In one patient, at the end of radiotherapy, gemcitabine was continued as maintenance. The others 8 patients received only gemcitabine at the same doses above-mentioned without radiotherapy. The study was approved by the hospital ethical committee. Tissue specimens were immediately frozen in liquid nitrogen, and stored at −80°C until RNA extraction. All the patients signed the informed consent before tissues collection. Demographics and clinical-pathological characteristics of patients are listed in Table 1.

**Table 1:** **Clinical and pathological features of 26 patients with Pancreatic Ductal Adenocarcinoma treated with Gemcitabine after pancreatic resection**

Age at diagnosis (years), median (Q1-Q3)	64.5 (51–73)
Gender, male/female (%male)	17/9 (65)
Smoking habit, n (%)	
No	10 (48)
Yes	8 (38)
Ex	3 (14)
Missing information	5
Alcohol Use, n (%)	
No	18 (95)
Yes	1 (5)
Missing information	7
Jaundice, y/n (%y)	15/11 (58)
Diabetes mellitus, y/n (%y)	9/17 (35)
Familial*, y/n (%y)	5/21 (19)
Previous Neoplasia, y/n (%y)	2/24 (8)
Preoperative serum CAE levels (ng/ml), median (Q1-Q3)	3.2 (2.0-6.0)
Preoperative serum CA 19–9 levels (U/ml), median (Q1-Q3)	200.4 (53.5-335.9)
Size (cm), median (Q1-Q3)	3 (2.4-3.5)
Tumour type, n (%)	
Adenocarcinoma	22 (85)
Adenocarcinoma Mucinous	4 (15)
Tumour grading, n (%)	
G1: well differentiated	5 (20)
G2: moderately differentiated	10 (40)
G3: poorly differentiated	10 (40)
Missing information	1
T: Tumour size, n (%)	
T1	1 (4)
T2	2 (8)
T3	23 (88)
N: regional lymph nodes, n (%)	
N0	3 (12)
N1	23 (88)
Lymph nodes ratio, median (Q1-Q3)	0.24 (0.07-0.47)
Tumour stage, n (%)	
IIA	3 (12)
IIB	23 (88)
Perineural Invasion, y/n (% y)	14/12 (54)
Vascular Invasion, y/n (% y)	3/23 (12)
Margins of resection, n (%)	
R0: negative resection margins	18 (69)
R1: microscopic positive resection margins	8 (31)
Postoperative serum CAE levels (ng/ml), median (Q1-Q3)	3.4 (2.0-4.5)
Postoperative serum CA 19–9 levels (U/ml), median (Q1-Q3)	10.4 (7.3-36.2)
Treatment with Gemcitabine	
Nr. Cycles, median (Q1-Q3)	5 (3–7)
Nr. Cycles < 6, n (%)	10 (39)
Nr. Cycles > 6, n (%)	16 (62)
Overall Follow-up (yrs), median (Q1-Q3)	1.4 (0.9-2.3)
Disease progression Follow-up (yrs), median (Q1-Q3)	0.9 (0.6-1.3)
Mortality rate**	16/45 (35)
Disease Progression rate**	17/29 (58)

*For neoplasia or chronic pancreatitis; **Number of events/person-years (expected number of events per 100 person-years).

### RNA isolation

Cryostat representative sections of the tumor were morphologically evaluated and neoplastic cellularity was enriched by microdissection of the most cellular areas. Total RNA was extracted from fresh frozen specimens by means of TRIzol® Reagent (Invitrogen, Milano, Italy) and subsequently purified using RNeasy®Mini Kit and digestion with DNase I. (Qiagen, Milano, Italy), according to manufacturer’s recommendations. RNA concentration and purity (A260:A280 > 2.0; A260/A230 > 1.8) were controlled by NanoDrop Spectrophotometer (Thermo Fisher, Waltham, MA, USA).

### Quantitative Real Time PCR

Expression analyses were performed using QuantiFast Sybr Green PCR kit (Qiagen, Milano, Italy), following the one-step protocol: cDNA was first synthesized from 60 ng total RNA, and then amplified by means of the Sybr Green QuantiTect Primer (Qiagen, Milano, Italy): *hENT1* (QT010000083), *CHOP* (QT00082278), *MRP1* (QT00061159) and *DKC* (QT00000392). Reactions were set up in 96-well plates and loaded onto 7700 Real-Time PCR System (Applied Biosystems, Foster City, CA). Optical data obtained were analyzed using the SDS software package (version 1.9.1; Applied Biosystems, Foster City, CA). Expression levels of target gene were obtained using the comparative method of relative quantification, after normalization for the housekeeping control gene Glyceraldehyde-3-phosphate dehydrogenase GAPDH (Sigma Aldrich, Milano, Italy), as previously performed [20].

### Immunohistochemistry

Formalin-fixed, paraffin-embedded PDAC sections were immunostained as already described [21] by using iVIEW DAB Detection Kit for Ventana BenchMark XT automated slide stainer on human biopsies. Primary antibodies for *hENT1* was purchased from Santacruz (cat. n. sc-134501) and diluted 1:100. Appropriate positive controls, as well as non-immune serum for negative controls, were run concurrently.

Normal pancreatic tissue samples were obtained from OriGene (Rockville, MD, USA) http://www.origene.com/tissue/tissue_qc.aspx. hENT1 immunoreactivity was evaluated in blind by two expert pathologists (FR and FC) assessing a semiquantitative scoring system in ten high power fields (10HPF, X 400) according to a semiquantitative scale (−: 0%; +: 1-33%; ++: 34-66%; +++: 67-100%).

### Statistical methods

Baseline patients’ characteristics were reported as frequency (percentages) and mean ± standard deviation (SD), along with median and lower (Q1) and upper (Q3) quartiles range, for categorical variables and continuous variables, respectively. Normal distribution assumption was checked by means of Q-Q plot, Shapiro-Wilks and Kolmogorov-Smirnov tests. To assess the presence of down/over regulation of genes expression in tumors compared to normal samples, one-sample *t*-test was performed using logarithm-transformed gene expression values. Correlations between (log-transformed) gene expression levels were assessed estimating Pearson’s correlation coefficients (r), whereas comparisons between log-transformed gene expression levels and categorical clinical variables were assessed using two-sample *t*-test or ANOVA models, respectively. Time to disease progression was defined as the time between the date of the surgery (baseline) and the date of the first progression event. Time to death was defined as the time between the baseline and the date of death. For subjects who did not experience any event, time variable was defined as the time between the baseline and the date of the last available clinical follow-up.

Incidence rates for events (i.e. disease progression or death, separately) were calculated as the number of events divided by the estimated persons-years, and eventually multiplied by 100.

To evaluate interactions between *hENT1*, *CHOP*, *DCK*, *MRP1* genes only, and between *hENT1*, *CHOP*, *DCK*, *MRP1* genes along with all patients’ clinical variables, identifying distinct and homogeneous subgroups of patients in terms of progression-free survival (PFS) and overall survival (OS), the RECursive Partitioning and AMalgamation (RECPAM) method was used [22,23]. The tree-growing algorithm estimates hazard ratios (HR), along with 95% confidence interval (95% CI), from a Cox proportional hazards regression model using appropriate covariates, as candidate splitting variables.

At each partitioning step, the method chooses the covariate and its best binary split to maximize the difference in the outcome of interest (i.e. PFS or OS). The algorithm stops when user defined conditions (stopping rules) are met (i.e. at least one event and at least three subjects per leaf). To obtain more robust and stable split (cut-off), a permutation approach was adopted to choose the best splitting variable. Furthermore, survival curves were drawn, for each final RECPAM class, from Cox proportional hazard models.

Moreover, all clinical features which could affect PDAC risk and clinical outcome of patients (i.e. diabetes mellitus, family history for neoplasia, tumor stage, positive surgical margins of resection, presence of vascular invasion, number of cycles of treatment with gemcitabine.) were compared between final RECPAM classes, using the Kruskal-Wallis and Fisher exact tests (due to non-normal data distribution and small sample size) for continuous and categorical variables, respectively.

A p value <0.05 was considered for statistical significance. All analyses were performed using SAS Release 9.3 (SAS Institute, Cary, NC, USA). For the RECPAM analysis a SAS macro routine, written by one of the authors (F. Pellegrini), was used.

## Results

### Relative expression levels of hENT1, CHOP, MRP1 and DCK in PDAC biopsies

**Figure 1:**
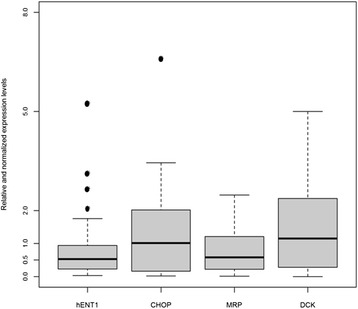
**Boxplot of relative expression levels of** ***hENT1*** **,** ***CHOP*** **,** ***MRP1*** **and** ***DCK*** **in matched pairs of tumor and normal samples from patients with Pancreatic Ductal Adenocarcinoma**. Each box highlights median (horizontal black bar), interquartile range (Q1-Q3) and lower and upper adjacent values (vertical bars) for each gene. Genes expression values are reported in log scale (y-axis). *p < 0.001.

Looking at median of gene expression levels in tumors compared to adjacent normal tissues, *hENT1* down-regulation in tumor samples was found (fold-change = 0.53, Q1-Q3 = 0.23-0.94, p = 0.006), (Figure 1). No differences in *CHOP, MRP1* and *DCK* expression levels were observed in tumors compared to normal tissues (*CHOP*: fold-change = 1.02, Q1-Q3 = 0.16-2.02, p = 0.266; *MRP1*: fold-change = 0.58, Q1-Q3 = 0.22-1.21, p = 0.203; *DCK* fold-change = 1.15, Q1-Q3 = 0.28-2.36, p = 0.370) Figure 1. Furthermore, *MRP1* expression levels were significantly correlated with those of both *hENT1* (r = 0.53, p = 0.006) and *CHOP* (r = 0.42, p = 0.032).

### Associations with clinical phenotypes

To establish whether mRNA levels were associated with specific diseases phenotypes, we analyzed the possible associations of genes expression with age at diagnosis, gender, smoking habit, alcohol use, presence of jaundice, diabete mellitus, family history for neoplasia, serum levels of carcinoembryonic antigen (CEA), tumor markers, tumor grading, tumor size, tumor histological type, lymph node spreading, staging of tumor, resection margins and medical therapy. In details, levels of *hENT1* were associated with jaundice and the resection margins of patients at diagnosis. hENT1 resulted differentially expressed in jaundiced patients compared to those without jaundice (jaundiced: fold-change = 0.61, Q1-Q3 = 0.32-1.76 *vs* not jaundiced: fold-change = 0.25, Q1-Q3 = 0.11-0.84; p = 0.047), and was differentially expressed in patients with evidence of tumor infiltration of the resection margins (R1) with respect to those with resection margins free from tumor cells (R0), (R1: fold-change = 1.35, Q1-Q3 = 0.32-2.37 *vs* R0: fold-change = 0.51, Q1-Q3 = 0.21-0.77; p = 0.030) showing a trend without reaching a statistical significance for the age (*hENT1*: r = 0.36, p = 0.067). *CHOP* resulted down-regulated in patients with family history of cancer (F1: fold-change = 0.11, Q1-Q3 = 0.09-0.16 *vs* F0: fold-change = 1.23, Q1-Q3 = 0.24-2.39; p = 0.044) and over-expressed in patients who had a previous neoplasia (PN1: fold-change = 31.77, Q1-Q3 = 1.57-61.98 *vs* PN0: fold-change = 0.66, Q1-Q3 = 0.13-1.93; p = 0.033). Interestingly, expression levels of *DCK* were found down regulated in mucinous adenocarcinoma (AM: fold change = 0.36, Q1-Q3 = 0.14-0.57 *vs* A: fold change = 1.34 Q1-Q3 = 0.34-2.94 p = 0.016). Finally, *MRP1* expression did not show any statically significant association with the clinical phenotypes taken in consideration.

### Survival analysis

In the overall sample, 16/26 (61.5%) patients died during the follow-up and 17/26 (65.3%) had a disease progression. Specifically, the overall mortality rate was 35 events per 100 person/years (median follow-up of 1.4 years), whereas the overall disease progression rate was 58 events per 100 person/years (median follow-up of 0.9 years).

**Figure 2:**
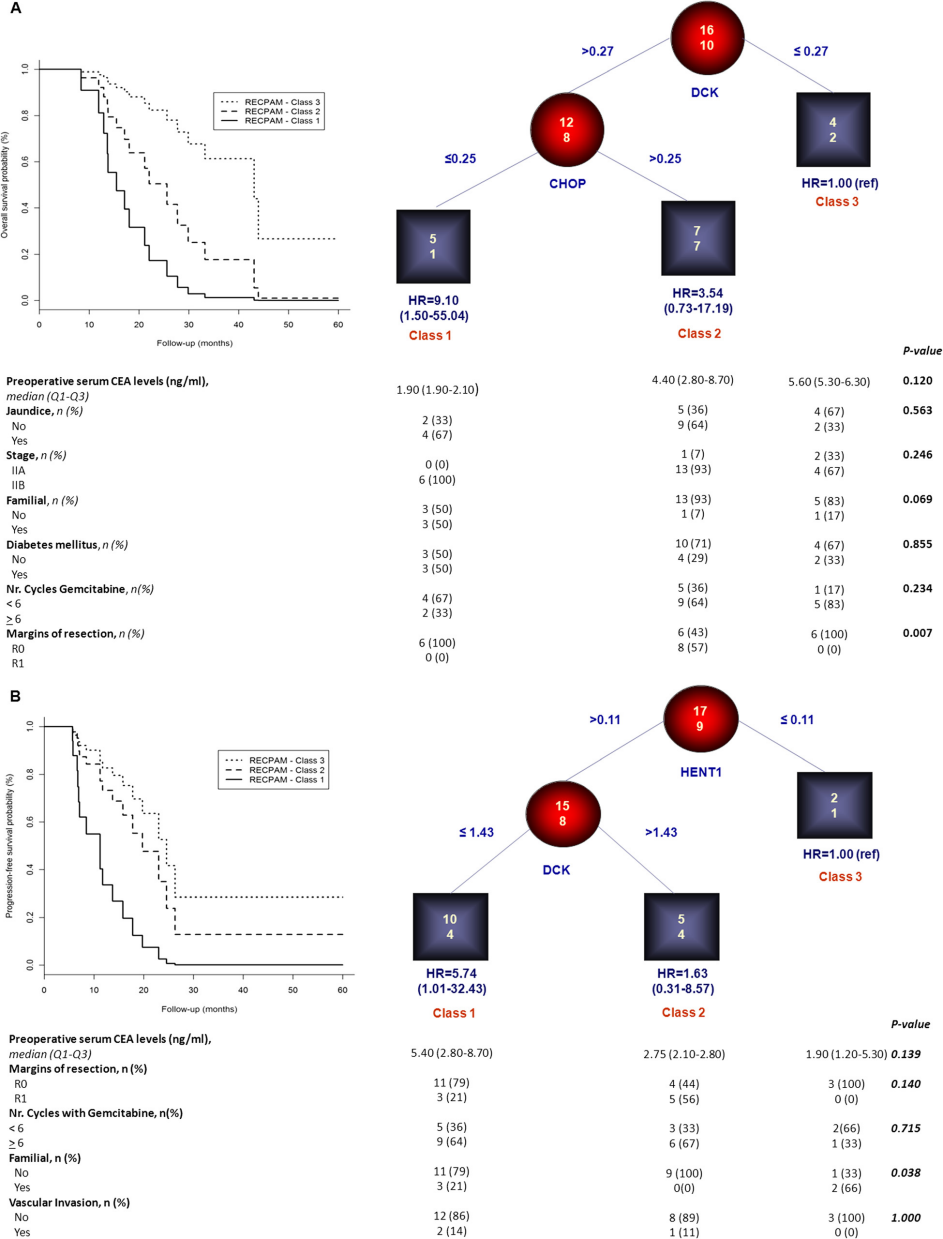
**Identification of subgroups at different risks based on gene-expression interactions: results of RECPAM analysis. A**: Classes of patients with different mortality risks; **B**: Classes of patients with different disease progression risks. Chosen splitting variables are shown between branches, while condition sending patients to left or right sibling is on relative branch. Circles indicate subgroups of patients. Squares indicate RECPAM classes. Numbers inside circles and squares represent the number of events (top) and the number of non-events (bottom), respectively. The table placed at the bottom of the figures **A**-**B** shows patients’ characteristics within each RECPAM class. Plot of survival curves, estimated from Cox proportional-hazards models, with respect to each identified RECPAM class for overall survival (panel A) and progression-free survival (panel B).

Focusing on the investigation of interactions between hENT1, CHOP, MRP1 and DCK genes on OS and PFS outcome, RECPAM tree-growing algorithm identified three classes at different mortality risks. As shown in Figure 2A, the reference class (Class 3) is represented by the subgroup with the lowest mortality, and all the HRs are estimated with respect to this reference class. In details, patients with DCK values ≤0.27 (n = 6) identified the reference class (HR = 1), whereas those with DCK > 0.27 and CHOP ≤ 0.25 (n = 6) identified the class with the highest mortality risk (Class 1, HR = 9.10, 95% CI = 1.50-55.04). Furthermore, patients with DCK > 0.27 and CHOP > 0.25 (n = 14) identified the intermediate risk class (Class 2, HR = 3.23, 95% CI = 0.64-16.23). The overall mortality rates (reported as the number of events per 100 person-years), along with median follow-up time, were: 63 (16.3 months), 35 (13.7 months), 23 (35.4 months) for Classes 1,2 and 3, respectively. (Figure 2A).

When looking at the clinical pathological features of patients within each RECPAM classes (please see profiles at the bottom of Figure 2A), we found that pre-operative serum levels of CEA were higher in patients with lowest mortality risk (and vice versa) while, although not statistically significant, the percentage of subjects with jaundice and stage IIB-cancer patients was slight higher within intermediate and highest mortality risk classes. Interestingly, half of the patients with highest mortality risk had family history of cancer and were affected from diabetes mellitus, whereas about two-thirds of them were treated with gemcitabine for less than six cycles. On the other hand, a significantly higher percentage of patients with positive surgical margins were observed within intermediate risk class, as compared to the other classes (p = 0.007).

Moreover, RECPAM algorithm also identified three classes at different disease-progression risk: as shown in Figure 2B, patients with *hENT1* ≤ 0.11 (n = 3) identified the reference class (HR = 1), whereas those with *hENT1* > 0.11 and *DCK* ≤ 1.43 (n = 14) identified the class with the highest disease-progression risk (Class 1, HR = 5.74, 95% CI = 1.01-32.43). Furthermore, patients with *hENT1* > 0.11 and *DCK* > 1.43 (n = 9) identified the intermediate risk class (Class 2, HR = 1.63, 95% CI = 0.31-8.57). Disease progression rates (reported as the number of events per 100 person-years), along with median follow-up time, were: 90 (7.7 months), 48 (11.2 months), 26 (23.0 months) for Classes 1,2 and 3, respectively. Figures 2A-B reports survival curves, identified by RECPAM analyses for OS and PFS, respectively.

When looking at the clinical pathological features of patients within each RECPAM classes (please see profiles at the bottom of Figure 2B), we found that patients with positive surgical margins belonged to Class 1 and 2, although no statistically significance was observed.

**Figure 3:**
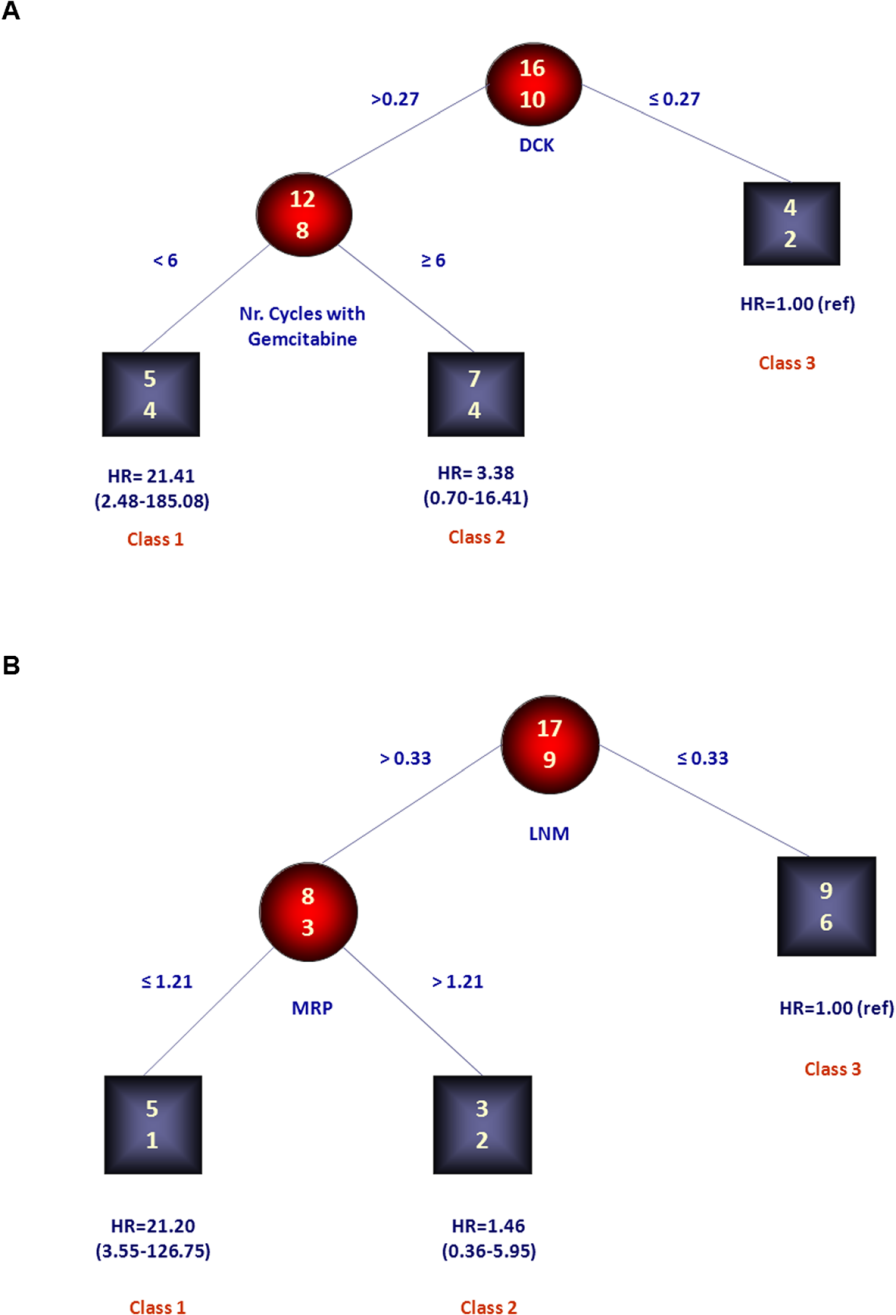
**Identification of subgroups at different risks based on patients’ clinical features-gene expression interactions: results of RECPAM analysis. A**: Classes of patients with different mortality risks; **B**: Classes of patients with different disease progression risks. Chosen splitting variables are shown between branches, while condition sending patients to left or right sibling is on relative branch. Circles indicate subgroups of patients. Squares indicate RECPAM classes. Numbers inside circles and squares represent the number of events (top) and the number of non-events (bottom), respectively.

When clinical variables were also included as candidate splitting variables, along with *hENT1, CHOP, DCK, MRP1* genes, RECPAM tree-growing algorithm identified three classes at different mortality risks: as shown in Figure 3A, patients with *DCK* ≤ 0.27 (n = 6) identified the reference class (HR = 1), whereas those with *DCK* > 0.27 and received <6 cycles of gemcitabine (n = 9) identified the class with the highest mortality risk (Class 1, HR = 21.41, 95% CI = 2.48-185.08). Furthermore, patients with *DCK* > 0.27 and received >6 cycles of gemcitabine (n = 11) identified the intermediate risk class (Class 2, HR = 3.38, 95% CI = 0.70-16.41).

Similarly, RECPAM tree-growing algorithm identified three classes at different disease-progression risks: as shown in Figure 3B, patients with lymph nodes ratio (LNM, i.e. the total involved lymph nodes among the total number of resected lymph nodes) ≤0.33 (n = 15) identified the reference class (HR = 1), whereas those with LNM >0.33 and MRP1 levels ≤ 1.21 (n = 6) identified the class with the highest disease-progression risk (Class 1, HR = 21.20, 95% CI = 3.55-126.75). Furthermore, patients with LMN > 0.33 and MRP1 levels > 1.21 (n = 5) identified the intermediate risk class (Class 2, HR = 1.46, 95% CI = 0.36-5.95).

### Immunohistochemistry

**Figure 4:**
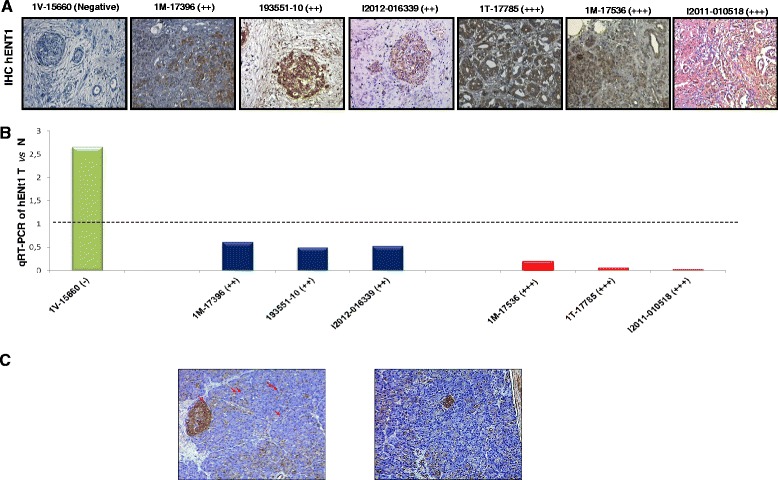
**Inverse correlation between hENT1 protein and mRNA expression. A**: Representative pictures of immunostainings performed for *hENT1* protein in samples of randomly selected pancreatic ductal adenocarcinomas. The immunohistochemical results are indicated in a semiquantitative scale (−: 0%; +: 1-33%; ++: 34-66%; +++: 67-100%). Original magnifications: 400X. **B**: *hENT1* mRNA expression levels in randomly selected pancreatic adenocarcinomas. **C**: representative pictures of immunostaining performed for hENT1 protein in control pancreatic tissue.

In order to perform a pilot evaluation of hENT1 protein localization/expression, immunohistochemical evaluations were carried out on 13 out of the 26 PDAC specimens used in the previous transcription analysis. Figure 4A and B shows 7 out of 13 selected representative immunoistochemistry of cases with highest *hENT1* mRNA (1 V-15660), intermediate (1 M-17396; 193551–10 and I2012-016339) or lowest levels (1 T-17785; 1 M17536 and I2011-010518) among all PDAC samples. Pathological features of these samples were listed in Table 2. hENT1 was mainly localized in the cytoplasm of the tumor cells. In particular, the strongest epithelial cell immunopositivity was observed in samples displaying the lowest hENT1 mRNA expression. By contrast, samples with an intermediate *hENT1* mRNA expression showed a lower positivity for hENT1 protein that was present in approximately a half of the cancer cells. Finally the only sample with the highest *hENT1* mRNA expression (1V-15660) was negative for hENT1 protein immunopositivity (Figure 4A-B). Strikingly, an inverse correlation between *hENT1* mRNA and protein levels was found. In fact, tissue samples from PDAC patients with higher levels of hENT1 mRNA displayed lower levels of the protein and, conversely, PDAC samples with low hENT1 protein levels showed higher levels of mRNA (Figure 4B). Noteworthy, normal pancreatic tissue displayed low hENT1 immunopositivity in the cytpoplasm and occasionally in the nuclei of the exocrine component (as indicated by the arrows in figure 4C) whereas high levels of hENT1 protein were observed in either cytoplasm and nuclei of the endocrine component.

**Table 2:** **Pathological features of 7 patients with Pancreatic Ductal Adenocarcinoma included in the immunohistochemistry analysis**

	**Age**	**Gender**	**Tumour type**	**Tumour Size (cm)**	**Tumour Grading**	**Tumour stage**	**Margins of resection**
**1 V-15660**	78	m	PDAC	3.05	G2-G3	IIB	R1
**1 M-17396**	69	m	Mucinous PDAC	3	G1	IIB	R0
**1 M-17536**	53	f	PDAC	3	G2-G3	IIA	R0
**1 T-17785**	65	m	PDAC	3	G2-G3	IIB	R0
**193551-10**	73	m	PDAC	1,8	G1	IIB	R0
**I2011-010518**	51	f	PDAC	2,5	G2	IIB	R0
**I2012-016339**	78	f	PDAC	2,4	G2	IIB	R0

G1: well differentiated; G2: moderately differentiated; G3: poorly differentiated. R0: negative resection margins; R1:microscopic positive resection margins. Tumor stage sec. AJCC 2010.

We propose the existence of a post-transcriptional feedback mechanism within PDAC cells that finely and reciprocally tune mRNA and protein levels of hENT1. In this respect, high levels of *hENT1* mRNA in the most aggressive tumors could be part of a compensatory and adaptive mechanism. Mechanistic studies are warranted to support this hypothesis.

## Discussion

Gemcitabine still represents the most common chemotherapeutic agent in the treatment of patients with PDAC [5,6]. However, nowadays cellular resistance to gemcitabine treatment represents the major problem in the clinical management of these patients [4]. Therefore, potential strategies to overcome and/or forecast chemoresistance are required in order to improve patient survival. In the present study, we examined gemcitabine related genes in a cohort of patients with PDAC underwent surgery and subsequent adjuvant treatment. To date a number of studies have evaluated *hENT1* and other factors involved in gemcitabine pathway by means of functional assays in pancreatic cancer cell lines and/or human specimens mainly by using immunoistochemistry, and transcriptional analysis in few cases [24-28]. Anyhow, our study represents the first attempt to concurrently analyze, in a well selected population of PDAC patients, mRNA expression levels of *hENT1* and other genes involved in gemcitabine pathway. In the overall sample we found a down-regulation of *hENT1* and *MRP1* in tumor compared to normal tissues, even though only *hENT1* down-regulation was statistically significant. On the other hand a substantially unchanged expression of *DCK* and *CHOP* emerged in our samples. Data on *DCK* were in line with those of Giovannetti and colleagues [28]. However, in this study, the authors found that *hENT1* was over-expressed in tumor specimens from patients with pancreatic cancer. This difference could be explained by the different number of subjects considered in the two studies, as well as by the pathological characterization of clinical specimens and the surgical/oncological treatment of patients.

In addition a direct correlation between levels of both *hENT1* and *CHOP* with *MRP1* expression emerged. These data suggest that the role of *hENT1* as main mediator of gemcitabine across plasma membrane could be also affected from expression of *MRP1* in PDAC tumor tissues, directly or as result of *CHOP* transcriptional regulation. In relation to patient clinical phenotypes the most interesting findings concern differential expression levels of *hENT1* with respect to the status of resection margins, a clinical pathological feature well recognized as prognostic factor of PDAC [29,30]. On the other hand, a significant association between hENT1 and jaundice at the diagnosis was observed. Jaundice represents the most common clinical symptom, albeit belated, of the disease, and has been also recently unraveled as a risk factor for diminished survival in patients with adenocarcinoma of the head of the pancreas [29]. Jaundice represents the most common clinical symptom, albeit belated, of the disease, and has been also recently unraveled as a risk factor for diminished survival in patients with adenocarcinoma of the head of the pancreas [31]. Significant correlations were also found for *CHOP* with respect to previous neoplasia and post-operative serum levels of CEA, one of the most common tumor markers used for clinical diagnosis of gastrointestinal and pancreatobiliary malignancies [32,33]. Finally, *DCK* down-regulation was associated with mucinous histotype of PDCA. Expression levels observed for the enzyme involved in gemcitabine phosphorylation/activation could explain the lower response to treatment observed in patients with mucinous disease compared to those without this pathological feature [34].

Several authors stated that higher *hENT1* levels were associated with significantly longer overall survival and disease free survival in patients affected by pancreatic cancer [25-28]. As we mentioned above, the disagreement with data of Giovannetti and colleagues [28] may be due to the differences in patients enrolled in the two studies. Moreover in our cohort, *hENT1* mRNA expression was inversely correlated with the protein expression levels and this also could explain the discrepancy observed in the literature among the different studies [14,28,35]_._ Our results, together with the previous observations, highlights that *hENT1* protein expression would be the most appropriate predictive marker.

Nevertheless, in order to determine the usefulness of intratumoral expression of *hENT1* and other factors involved in gemcitabine pathway as predictive markers of the efficacy of adjuvant gemcitabine-based chemotherapy for PDAC after operative resection, we used the RECPAM model to choose the natural cut-off points of genes expression levels for identification of patients at different-risk of mortality and disease progression. RECPAM analysis uncovered *DCK* and *CHOP* as the most important genes in distinguish patients with different mortality risk, while *hENT1* and *DCK* were able to identify subgroups of patients with different disease progression risk. Using these RECPAM models to evaluate the possible effect/contribution of patient clinical profiles on gene expression alterations, we also tested a set of variables made of the suggested risk or prognostic factors for PDAC.

## Conclusion

In this study we found that intermediate mortality risk was associated with positive surgical resected margins, whereas class of patients with high mortality risk was characterized by higher percentage of patients with different PDAC risk factors (family history). In relation to disease progression we showed that family history of neoplasia was associated. Our data suggested an interaction between *DCK* levels and the preoperative serum CEA levels and between preoperative serum CEA levels and tumor grading in relation to mortality and disease progression risk, respectively. Taken together all these data may help clinicians to classify disease behavior in PC patients, based on this gene expression pattern background.

In this study we found that intermediate mortality risk was associated with positive surgical resected margins, whereas class of patients with high mortality risk was characterized by higher percentage of patients with different PDAC risk factors (family history for neoplasia although not significant). In addition, classes of patients with either intermediate or high risk of PDAC showed both to be characterized by an higher trend of jaundice and by tumors stage and unsuccessful gemcitabine induction phase. Indeed, also jaundice has been recently described as important, although poorly recognized risk factor for diminished survival in patients with adenocarcinoma of the pancreas [29]. Similarly, in relation to disease progression we showed that both positive resection margins and unsuccessful gemcitabine induction phase were present within the classes of patients at higher and intermediate risk although no statistical significance was observed. Finally, our data suggested an interaction between DCK levels and the number of cycles of gemcitabine and between lymph nodes metastases and MRP1 expression in relation to mortality and disease progression risk, respectively. Taken together all these data may help clinicians to classify disease behavior in PC patients, based on this gene expression pattern background.
